# HIV Viral Load Trends in Six Eastern Caribbean Countries Utilizing a Regional Laboratory Referral Service: Implications for Treatment as Prevention

**DOI:** 10.1371/journal.pone.0125435

**Published:** 2015-04-29

**Authors:** R. Clive Landis, Kelly Carmichael-Simmons, Ian R. Hambleton, Anton Best

**Affiliations:** 1 Chronic Disease Research Centre, The University of the West Indies, Cave Hill, Barbados; 2 Ladymeade Reference Unit, Ministry of Health, St. Michael, Barbados; University of Alabama at Birmingham, UNITED STATES

## Abstract

**Objective:**

Since 2009, seven countries in the Organization of Eastern Caribbean States (OECS), Antigua & Barbuda, Dominica, Grenada, Montserrat, St. Kitts & Nevis, Saint Lucia, and St. Vincent & the Grenadines, have been utilizing a laboratory referral service for HIV-1 viral load (VL) offered by The Ladymeade Reference Unit (LRU) Laboratory, Barbados. The objective of this study was to evaluate 5 year VL trends in the six larger OECS countries participating in this regional referral service.

**Methods:**

Blood samples were collected in source countries and transported to Barbados as frozen plasma according to a standardized protocol. Plasma specimens were amplified by RT PCR on a Roche TaqMan 48 analyser (Roche Diagnostics, Panama City, Panama). VL was considered optimally suppressed below a threshold level of < 200 HIV-1 copies/mL of blood. The same threshold was used as a binary indicator in an analysis of the secular change in VL suppression. Montserrat was excluded due to insufficient number of samples.

**Results:**

A steady rise in VL referrals from OECS countries was recorded, rising from 312 samples in 2009 to 1,060 samples in 2013. A total of 3,543 samples were tested, with a sample rejection rate (9.2%) mostly due to breaks in the cold chain. Aggregate VL data showed the odds of VL suppression in the Eastern Caribbean improved by 66% for each additional year after 2009 (Odds Ratio 1.66 [95% CI 1.46 to 1.88]; p<0.001).

**Conclusion:**

We demonstrate the feasibility of establishing a regional laboratory referral service for HIV VL monitoring in the Eastern Caribbean. Aggregate VL trends showed a significant year-on-year improvement in VL suppression, implying public health benefits through treatment as prevention in the OECS. VL provides a powerful monitoring & evaluation tool for strengthening HIV programs at country level among the small island states participating in this regional referral network.

## Introduction

The Caribbean has the second highest HIV prevalence in the world (0.9%) outside that of sub-Saharan Africa, with 200,000 persons estimated to be living with HIV [1]. HIV/AIDS remains the leading cause of death in the 25–44 age group in the Caribbean [2]. The Organization of Eastern Caribbean States (OECS) is a nine member group of small island states with a combined population of approximately 600,000 bound by a treaty of cooperation signed in 1981 [3]. Individual member state populations in 2010 range between 5,000 for Montserrat to 177,000 for Saint Lucia. The seven states that participated in the laboratory referral service are: Antigua & Barbuda, The Commonwealth of Dominica, Grenada, Montserrat, St. Christopher (St. Kitts) & Nevis, Saint Lucia, and St. Vincent & the Grenadines. The estimated adult HIV prevalence in the OECS is reported by the Pan American Health Organization (PAHO) at 0.75% in Dominica, 0.9% in St. Kitts & Nevis and as “not available” for the remaining five OECS countries [4]. USAID reports adult HIV prevalence among OECS countries as “under 1%” [5].

There is much interest in the HIV field on the public health benefits of antiretroviral therapy, a paradigm known as “Treatment as Prevention”. Cohort studies, critical reviews and randomized control trials have all suggested that sexual transmission of HIV is virtually eliminated in persons receiving ART with demonstrated VL suppression [6–10]. Treatment as Prevention has been accepted by policymakers as an important public health tool [11;12] and lies at the heart of the WHO consolidated guidelines on the use of antiretroviral drugs for treating and preventing HIV infection [13]. VL monitoring is the metric of choice to evaluate the effectiveness of patient engagement in the continuum of care and has been adopted into the regional monitoring framework for HIV prevention by PAHO [13–15].

A threshold value for optimal VL suppression has been proposed by the Centers for Disease Control and Prevention (CDC) at ≤200 copies/ml [16]. Using this threshold value, the CDC estimated 28% of the HIV infected population in the USA had optimally suppressed VL [17]. By the same methodology, viral suppression in Barbados was 26% of the HIV infected population [18]. Similar information is not available from other Caribbean countries, partly because of logistical challenges in VL testing.

Many small island developing states lack the necessary infrastructure to conduct the VL test, despite its recognized role in the monitoring of HIV treatment programs [14;19]. None of the OECS countries has in-country access to this test. The current state of technology depends on high throughput instruments typically housed in reference laboratories. VL testing was introduced in the Ladymeade Reference Unit (LRU) Laboratory in Barbados in 2002 and the laboratory has been internationally accredited by the College of American Pathologists (CAP) since 2009 [20]. A laboratory referral network among Eastern Caribbean states with Barbados as the hub was envisaged from meetings coordinated in 2008 by the Caribbean MedLabs Foundation and the Caribbean Cytometry & Analytical Society [21]. Since 2009 the LRU laboratory has offered a VL referral service to the OECS countries.

Here we review uptake of the VL referral service and report five year trends of VL suppression in OECS countries between 2009–2013.

## Methods

### Patient population

A total of 3,543 coded patient samples were received by The Ladymeade Reference Unit (LRU) Laboratory from OECS countries over a five year period 2009–2013. Samples were sent as frozen plasma in batches from national HIV treatment centres or from treating physicians in OECS countries. Anonymized viral load data was aggregated by year and country for analysis. The secondary analysis of aggregated data performed in this study from a coded dataset containing no personal identifiers was waived by the chairman of the joint Ministry of Health/University of the West Indies Ethical Review Board (ERB) from further consideration of the ERB. The Ministry of Health does not require explicit consent from patients accessing healthcare facilities for permission to use aggregate laboratory indicators collected as part of routine patient care for the purposes of disease surveillance or programmatic monitoring and evaluation. This stance is supported by the joint Ministry of Health/University of the West Indies Ethical Review Board.

### Blood collection and shipping

Two 4ml samples of EDTA anticoagulated blood were collected from patients in source countries and plasma was separated by centrifugation at 800-1600xg for 20 minutes at room temperature (18–25°C). Centrifuged plasma was transferred into two sterile cryotubes, which were frozen at -80C prior to shipment in batches on ice. Samples were rejected by the receiving laboratory if they had warmed to ambient temperature, if there was any leakage, or if they were incorrectly identified. All aspects of blood collection and shipping were governed by a standard operating procedure (SOP) prepared by the LRU Laboratory: document ‘LRU-M02A-Shipping’.

### Viral Load Measurement

Viral load determinations were performed in Barbados using the Roche AmpliPrep and TaqMan 48 Analysers (Roche Diagnostics), with a lowest detection limit of 20 copies/ml.

### Statistical Analysis of Viral Load Trends

Many patients were tested repeatedly during the study period and we present the number of patients, the number of viral load measurements, and the average number of measurements per patient, stratified by country and year of measurement. We present log viral load summaries by country and year of measurement (median, interquartile range). The thresholds for VL suppression (≤ 200 copies/ml) was adopted from the CDC document: “Guidance on Community Viral Load: A Family of Measures, Definitions, and Method for Calculation” [16]. To examine secular VL change we fitted two random-effect logistic regression models with VL control (yes or no) as the outcome of interest. Each model accounted for the increased correlation of multiple measurements within an individual and was pre-adjusted for suppression differences between countries. First, we included year as a continuous model term to test for a linear trend in the proportion of controlled individuals (between 2009 and 2013). Second, we included year as a categorical term to examine the improvment in VL control in each study year compared to 2009. Exact p-values and 95% confidence intervals are presented when appropriate to clarify the strength of statistical relationships. Statistical analyses were performed using Stata statistical software (version 13, StataCorp., College Station, TX).

## Results

### Uptake of the laboratory referral service: geographic and demographic trends

**Table 1** shows the distribution of patients and samples tested across the laboratory referral network by country and by year. Antigua and St. Vincent collectively comprised 53% of participants and 50% of the submitted samples, with St. Lucia (22% and 20%), Grenada (12% and 12%), Dominica (7% and 12%), and St. Kitts & Nevis (6% and 7%) submitting the remaining samples. There were just 21 samples in total tested from Montserrat (data not shown), too few to include in a country level analysis. Among the six larger OECS countries there were a total of 3,543 samples tested from 1,654 patients between August 2009 and December 2013.

**Table 1:** Number of participants, number of viral load measurements, and average number of viral load measurements per participant, stratified by country and year of measurement.

YEAR	MEASURE	COUNTRY						
		Antigua	Dominica	Grenada	St.Kitts	St.Lucia	St.Vincent	ALL COUNTRIES
**2009**	**# participants**	81	38	20	20	116	6	281
** **	**# measurements**	96	45	20	21	124	6	312
** **	**mean measurements**	1.19	1.18	1.00	1.05	1.07	1.00	1.11
** **	**sd measurements**	0.39	0.39	—	0.22	0.29	—	0.33
**2010**	**# participants**	123	46	36	33	101	59	398
** **	**# measurements**	138	64	40	54	108	67	471
** **	**mean measurements**	1.12	1.39	1.11	1.64	1.07	1.14	1.18
** **	**sd measurements**	0.42	0.58	0.32	1.29	0.26	0.43	0.55
**2011**	**# participants**	138	64	66	21	128	135	552
** **	**# measurements**	148	100	81	33	140	143	645
** **	**mean measurements**	1.07	1.56	1.23	1.57	1.09	1.06	1.17
** **	**sd measurements**	0.26	0.66	0.42	0.93	0.29	0.24	0.43
**2012**	**# participants**	189	69	110	46	163	244	821
** **	**# measurements**	281	100	142	64	178	290	1,055
** **	**mean measurements**	1.49	1.45	1.29	1.39	1.09	1.19	1.29
** **	**sd measurements**	0.84	0.70	0.46	0.61	0.31	0.39	0.58
**2013**	**# participants**	205	71	103	45	144	292	860
** **	**# measurements**	262	118	123	59	157	341	1,060
** **	**mean measurements**	1.28	1.66	1.19	1.31	1.09	1.17	1.23
** **	**sd measurements**	0.54	0.70	0.42	0.56	0.29	0.40	0.48
**ALL YEARS**	**# participants**	446	120	201	93	368	426	1,654
** **	**# measurements**	925	427	406	231	707	847	3,543
** **	**mean measurements**	2.18	3.58	2.02	2.49	1.93	2.00	2.18
** **	**sd measurements**	1.74	2.80	1.38	2.64	1.17	1.20	1.69

From **Table 1** and across all years, Antigua submitted 925 samples from 446 patients (2.18 samples per patient), St.Vincent submitted 847 from 426 patients (2.00 samples per patient), St. Lucia 707 from 368 (1.93 samples per patient), Grenada 406 from 201 (2.02 samples per patient), Dominica 427 from 120 (3.58 samples per patient), and St. Kitts 231 from 93 (2.49 samples per patient). St. Vincent has evolved from being the smallest referral country in 2009 to being the largest in 2013.

From 3,543 samples, 325 (9.2%) were spoiled (mainly due to breaks in cold chain during shipping) and so were not tested, leaving 3,216 samples tested for viral load. Among tested samples, the sex of the patient was available for 2,957 (91.9%) of the samples, and the distribution of samples by country, year of test and sex is presented in **Table 2.** The data highlight a general male testing excess (6.4% more male samples), a particularly large male excess in Dominica (19.4% more male samples), but country variation in this pattern, with St. Lucia and St. Kitts & Nevis having more female samples (St. Lucia: 7.4% more female samples, St. Kitts & Nevis 11.1% more female samples).

**Table 2:** Percentage of viral load measurements by sex of patient, by country, and by year between 2009 and 2013.

YEAR	COUNTRY						
	Antigua	Dominica	Grenada	St.Kitts	St.Lucia	St.Vincent	ALL COUNTRIES
**WOMEN**							
**2009**	0.9	0.7	0.3	0.4	2.3	0.1	4.7
**2010**	1.1	0.8	0.6	0.6	1.8	0.6	5.5
**2011**	1.1	1.5	1.2	0.5	2.5	2.4	9.3
**2012**	3.0	1.1	1.6	1.1	2.1	4.4	13.3
**2013**	2.8	1.3	1.7	0.9	2.2	5.1	14
**ALL YEARS**	8.9	5.4	5.4	3.5	10.9	12.6	46.8
**MEN**							
**2009**	1.4	0.8	0.3	0.3	1.8	0.1	4.7
**2010**	1.4	1.0	0.7	0.4	1.8	1.2	6.5
**2011**	1.3	1.9	1.4	0.4	2.2	2.3	9.4
**2012**	4.0	2.0	2.5	0.7	1.9	5.0	16
**2013**	3.8	2.3	1.9	1.0	1.7	6.0	16.6
**ALL YEARS**	11.9	8.0	6.8	2.8	9.4	14.6	53.2
**% SEX DIFFERENCEMALE—FEMALE**	14.4	19.4	11.5	-11.1	-7.4	7.4	6.4

NOTE: The sex of the patient was available for 2,957 / 3,216 (91.9%) of the samples

### Five year viral load trends

**Fig 1:**
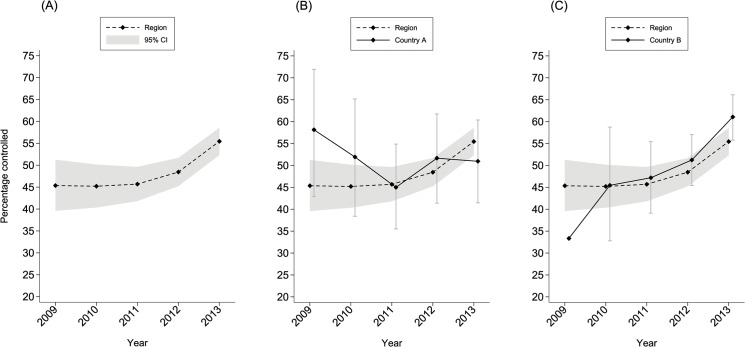
Viral load control among OECS countries. The proportion of samples achieving controlled VL (defines as < 200 copies/ml [16]) from six Eastern Caribbean countries between 2009 and 2013 (A). Examples of individual country reports for VL trends compared to the regional average are shown for (anonymized) countries A and B (B, C). The shaded area shows the 95% confidence intervals for the whole dataset. Error bars show the 95% confidence intervals for individual countries A and B.

The median (logged) VL counts across the OECS network decreased from 2.7 in 2009 to 1.8 in 2013 (**Table 3**). Using a threshold value of 200 copies/ml to define optimal virological suppression [16], the proportion of patients with suppressed VL improved from 45.4% in 2009 to 55.4% in 2013 (**Table 3** and **Fig 1A**). Assuming a linear effect of measurement year on VL suppression, the odds ratio for each one-year change was 1.66 (95% CI 1.46, 1.88) (p<0.001), meaning that the odds of VL suppression in the Eastern Caribbean increased by 66% for each additional year after 2009. Fig 1 highlights that the VL suppression effect is currently non-linear, and if we instead compare each measurement year against a 2009 baseline we note odds ratios of 1.07 (95% CI 0.58, 1.96) in 2010, 1.32 (95% CI 0.76, 2.31) in 2011, 2.50 (95% CI 1.44, 4.35) in 2012 and 5.94 (95% CI 3.32, 10.61) in 2013, reflecting increasing VL suppression and an acceleration of that suppression.

**Table 3:** Logged viral load and proportion of patients with suppressed VL among 3,216 samples collected between 2009 and 2013.

Year	N	Median log viral load	% VL suppression
		(copies/mL) (IQR)	(≤ 200 copies/mL)
2009	280	2.6 (1.6–4.4)	45.4
2010	396	2.9 (1.6–4.4)	45.2
2011	626	2.8 (1.3–4.5)	45.7
2012	931	2.5 (1.3–4.5)	48.4
2013	983	1.9 (1.3–4.2)	55.4
ALL YEARS	3,216	2.4 (1.3–4.4)	49.4

## Discussion

We have demonstrated that a viable VL laboratory referral service can be established in the Eastern Caribbean between seven OECS countries and a reference laboratory in Barbados. This referral service detected a statistically significant year on year trend towards improved VL suppression across the region, with implications for HIV prevention in member states. However, it must be noted that despite an overall improvement, individual countries showed variations, with some countries demonstrating no improvement and others reporting above trend improvements. Examples of such trends are illustrated in Fig 1 for countries A and B—without identifying the country by name in order to protect confidentiality agreements. Confidential reports are passed onto individual health ministries, providing evidence for each country’s performance with respect to the regional average, in order to strengthen programmatic monitoring and evaluation (M & E) within contributing countries.

The importance of VL suppression as a public health tool in the fight against HIV transmission cannot be overstated. The Caribbean has earned praise from UNAIDS for recording decreasing trends in new infections [22], with strong gains recorded in prevention of mother to child transmission (PMTCT) and treatment as prevention programs [18;23;24]. Declining infection rates are likely to be underpinned by the prevention benefits of ART, recognized by the WHO consolidated guidelines and the regional PAHO M & E framework that identifies viral load as the key metric for evaluating patient engagement in the continuum of care [13;14]. Community VL may even act as **a** proxy for the likelihood of future infection within a community [25;26]. A regional network for VL surveillance therefore provides a powerful M & E tool for HIV programs [15;19;27]. We anticipate that the temporal trends for VL suppression will provide OECS countries with objective markers of personal as well as public health outcomes in order to strengthen their national HIV responses.

The trends in sample referrals by each country are partly attributable to the size of the countries concerned and differential rates of uptake of the service. The OECS countries each demonstrate a greater proportion of males living with HIV than females, reflecting the trend of the epidemic in the Caribbean which began in men [1;2]. All available evidence suggests that increased uptake of the service in St. Vincent explains its rise from being the smallest referral country in 2009 to the largest in 2013.

Despite the quality assurance provided by international accreditation through CAP [20], there remain some concerns regarding the functioning of the referral service and the long term sustainability of the network. Nearly **9**% of samples had no designation of sex, suggesting problems in data collection or labeling of samples. In other concerns, a report commissioned by the Caribbean MedLabs Foundation (CMLF) in 2013 [28] noted that despite an overall positive satisfaction rating, a lack of human resources in Barbados had contributed to inconsistencies in turnaround time. The report also identified significant threats to financial sustainability from two sources: First, the service which operates on a cost recovery basis, is not in fact financially self-sustaining from the perspective of Barbados and entails a small loss each year borne by the Barbados Ministry of Health. Second, the funds used to support the service have been made available through extra-regional grants, initially a Global Fund grant and more recently a grant from the US President’s Emergency Program For AIDS Relief (PEPFAR). With prevailing trends showing a decline in HIV funding to the Caribbean from global institutions the long term sustainability of the referral service cannot therefore be taken for granted [21;29]. To mitigate against the uncertain funding situation, it has been proposed that laboratory services be incorporated into the Pharmaceutical Procurement Service of the OECS, a pooled purchasing programme that has been credited with bringing stability to the supply of ARVs to OECS countries [30].

There are some limitations to this study. While the reference laboratory is able to report aggregate VL trends for the purpose of programmatic strengthening within a country, comparisons between countries may not be valid. The anonymization process renders the reference laboratory blind to the sample source, which may derive from adults, children, antenatal screening, high risk groups, clients failing on HAART, or a combination of some or all of the above. For example, the male testing excess in Dominica or the female testing excess in St. Lucia may reflect different epidemic patterns across the islands, but they may equally represent differential testing regimes among healthcare providers or differences in patient groups. We also remain cautious interpreting the strength of temporal trends within countries, which may be confounded by relatively low sample numbers.

Nonetheless, the referral service has proven the principle that a Caribbean laboratory network is feasible and can thrive. It provides a template to encourage further expansion of laboratory services within the Caribbean region, particularly those utilizing expensive molecular platforms, and the LRU laboratory is already handling other regional referrals for early infant diagnosis for HIV by DNA PCR, HIV drug resistance testing, and molecular testing for *Chlamydia trichomonas* and *Neisseria gonorrhea* (CT/NG) [28]. This nascent laboratory network will in the future benefit from a strengthened governance structure being established under the aegis of the Caribbean Public Health Authority (CARPHA) to define laboratory reference services, standards and networks [31].

## Supporting Information

S1 Dataset A dataset of categorical variables has been provided (S1_Dataset.xls). This sensitive HIV dataset has been anonymized to protect the identity of patients and countries participating in the laboratory referral service.
(XLSX)
